# RETRACTION: A Novel *In Situ* Dendritic Cell Vaccine Triggered by Rose Bengal Enhances Adaptive Antitumour Immunity

**DOI:** 10.1155/jimr/9834758

**Published:** 2025-11-13

**Authors:** Journal of Immunology Research

RETRACTION: L. Zhang, J. Du, Q. Song, C. Zhang, and X. Wu, “A Novel *In Situ* Dendritic Cell Vaccine Triggered by Rose Bengal Enhances Adaptive Antitumour Immunity,” *Journal of Immunology Research* 2022 (2022): 1178874, https://doi.org/10.1155/2022/1178874.

The above article, published online on 1 February 2022 in Wiley Online Library (wileyonlinelibrary.com), has been retracted by agreement between the journal Editor‐in‐Chief, Nadja Bakocevic; and John Wiley & Sons Ltd.

The retraction has been agreed following an investigation of the concerns raised by *Indigofera tanganyikensis* on PubPeer [1], which identified an instance of figure duplication.

Specifically, the images of B‐16 and LLC cells treated with 100 μM of RB with isotype control in Figure 2a share unexpected similarities.

As a result of the investigation, the data and conclusions of this article are considered unreliable.

The authors have been informed of this retraction but did not provide a response.
